# Sustainability Consciousness, Green Advocacy, and Work Grit Among Nurses: Implications for Environmentally Sustainable Healthcare and Public Health

**DOI:** 10.3390/ijerph23040523

**Published:** 2026-04-17

**Authors:** Eman Kamel Hossny, Noura Alsayed Esmeil, Hanan Sayed Younes, Eman Ramadan Abdalfadeel, Ahmed Zinhom Elkady, Hammad S. Alotaibi, Somia Mohamed Abdel Aziz

**Affiliations:** 1 Community and Mental Health Nursing Department, Faculty of Nursing, Zarqa University, Zarqa 13132, Jordan; 2 Nursing Administration Department, Faculty of Nursing, Assiut University, Assiut 71515, Egypt; noura@aun.edu.eg (N.A.E.); hanan.sayed1990@aun.edu.eg (H.S.Y.); somia_mohamed@aun.edu.eg (S.M.A.A.); 3 Faculty of Nursing, Primary Nursing Department, Al-Ahliyya Amman University, Amman 19328, Jordan; e.ramadan@ammanu.edu.jo; 4 Nursing Administration Department, Faculty of Nursing, Cairo University, Cairo 11311, Egypt; 5 Nursing Department, North Private College of Nursing, Arar 73521, Saudi Arabia; ahmadzinhom@nec.edu.sa; 6 College of Taraba, Taif University, Taif P.O. Box 11099, Saudi Arabia; hammad@tu.edu.sa

**Keywords:** sustainable healthcare, environmental sustainability, green advocacy, sustainability consciousness, work grit, nurses

## Abstract

**Highlights:**

**Public health relevance—How does this work relate to a public health issue?**

- This study examines how nurses’ sustainability consciousness influences environmentally responsible practices in healthcare settings.
- It explores the role of work grit in strengthening nurses’ engagement in green advocacy that supports sustainable healthcare systems.

**Public health significance—Why is this work of significance to public health?**

- Healthcare systems significantly contribute to environmental impacts, making sustainability awareness among healthcare professionals a critical public health concern.
- Understanding the psychological and behavioral factors that promote green advocacy among nurses can support environmentally responsible healthcare practices.

**Public health implications—What are the key implications or messages for practitioners, policy makers and/or researchers in public health?**

- Strengthening sustainability education and organizational support may enhance nurses’ ability to advocate for environmentally responsible healthcare practices.
- Integrating sustainability competencies and resilience-building strategies into nursing training and policy initiatives may contribute to more sustainable healthcare systems.

**Abstract:**

Background: Healthcare systems contribute significantly to environmental pollution, energy consumption, and resource depletion, making sustainability an increasingly important environmental and public health priority. Nurses, as frontline healthcare professionals, play a critical role in promoting environmentally responsible practices and advocating for sustainable healthcare within clinical settings. Objective: The study aimed to examine the associations between nurses’ sustainability consciousness, green advocacy, and work grit in hospital settings. Methods: A descriptive cross-sectional correlational study was conducted among 377 nurses working in two university-affiliated hospitals in Egypt. Data were collected using validated instruments assessing sustainability consciousness, green advocacy, and work grit. Descriptive statistics were calculated to summarize participant characteristics and study variables. Associations among sustainability consciousness, green advocacy, and work grit were examined using Pearson correlation analysis. Multiple linear regression analysis was conducted to identify significant predictors of green advocacy, while noting that the study design allows for identification of associations rather than causal relationships. Results: The findings indicated generally high levels of sustainability consciousness among nurses. Significant positive associations were observed between sustainability consciousness, green advocacy, and work grit (*p* < 0.01). Multiple linear regression analysis identified sustainability consciousness and work grit as significant predictors of green advocacy, explaining 34.2% of its variance. Conclusions: These findings highlight the interconnected roles of sustainability awareness, advocacy behaviors, and psychological resilience in promoting environmentally sustainable healthcare practices. Strengthening nurses’ sustainability consciousness and work grit may enhance green advocacy and contribute to the development of sustainable healthcare systems, supporting global environmental and public health goals aligned with the United Nations Sustainable Development Goals.

## 1. Introduction

Healthcare systems are increasingly recognized as major contributors to environmental degradation due to their high consumption of energy and water, extensive use of single-use plastics, and substantial waste generation, all while striving to maintain high standards of patient care [1]. These environmental pressures place healthcare at the center of global sustainability challenges and underscore the urgent need for system-level transformation aligned with international sustainability agendas, including the United Nations Sustainable Development Goals (SDGs), particularly those related to good health and well-being, climate action, and sustainable communities [2].

Within this context, healthcare professionals play a critical role in advancing sustainable healthcare practices. Nurses, who constitute the largest segment of the healthcare workforce and are deeply embedded in daily clinical operations, occupy a strategic position within healthcare systems and sustainability transitions [3]. Through their continuous interaction with patients, colleagues, and organizational processes, nurses can influence environmentally responsible practices not only at the individual level but also across units, workflows, and institutional decision-making structures, thereby supporting organizational sustainability goals [4].

The healthcare sector accounts for approximately 5% of global greenhouse gas emissions, with hospitals representing the largest contributors and critical care units generating nearly three times the emissions of other hospital patient care areas. Environmental harm associated with healthcare delivery includes excessive medical waste, toxicant exposure, and pollutant emissions [5]. Although integrating green practices into hospital settings—particularly in resource-intensive environments such as intensive care units—can be challenging due to time constraints, limited leadership support, organizational resistance, and gaps in sustainability knowledge, nurses remain central to overcoming these barriers and facilitating the implementation of sustainable practices within complex healthcare systems [6].

A central concept underpinning sustainable engagement in healthcare is sustainability consciousness, which encompasses individuals’ awareness, knowledge, attitudes, and behaviors related to the environmental, social, and economic dimensions of sustainable development. Among nurses, sustainability consciousness has been associated with greater engagement in eco-friendly practices and an increased sense of responsibility for minimizing the environmental impact of healthcare delivery. However, achieving meaningful and sustained sustainability outcomes requires moving beyond individual behaviors toward collective and organizational action through green advocacy [2]. Green advocacy refers to nurses’ efforts to promote, support, and influence environmentally sustainable practices within healthcare organizations and the wider community, including education, behavioral encouragement, and organizational or policy-level changes to protect human and environmental health [7].

As healthcare systems confront the escalating health consequences of climate change, nurses are increasingly recognized as key contributors to environmental sustainability within resource-intensive care settings [2]. However, translating sustainability consciousness into consistent and sustained advocacy actions requires more than awareness alone. Personal psychological resources, particularly work grit, may play a critical enabling role in this process. Work grit—defined as perseverance, resilience, and sustained commitment despite workplace challenges—has been associated with sustained motivation, professional engagement, and adaptive coping among nurses in demanding healthcare environments [8]. Emerging evidence suggests that higher sustainability consciousness is linked to stronger work grit, enabling nurses not only to adopt environmentally responsible behaviors but also to persistently advocate for sustainable organizational and policy-level change [9].

When sustainability consciousness is reinforced by work grit, nurses may be better equipped to navigate organizational resistance, maintain long-term engagement in green advocacy initiatives, and contribute to sustainability transitions within healthcare systems [10]. In this sense, work grit functions as a psychological resource that strengthens the translation of sustainability awareness into sustained advocacy behaviors and supports the integration of sustainability principles into routine healthcare practice and organizational culture [11].

Despite the growing emphasis on sustainable healthcare, empirical research examining the combined roles of sustainability consciousness, green advocacy, and psychological resilience among nurses remains limited. Existing studies have primarily focused on sustainability awareness or environmentally responsible behaviors in isolation, with comparatively little attention given to how personal resilience factors such as work grit may support sustained advocacy and organizational-level sustainability change, particularly in resource-constrained healthcare settings [12]. Addressing this gap is essential for informing workforce development strategies and organizational policies aimed at fostering long-term sustainability and resilience in healthcare systems.

The conceptual framework proposes that sustainability consciousness among nurses serves as a foundational determinant that enhances their moral obligation toward environmental responsibility. This moral obligation, in turn, fosters green advocacy behaviors, which ultimately lead to sustained effort in implementing environmentally sustainable practices. Additionally, sustainability consciousness may directly influence green advocacy, while moral obligation may also contribute directly to sustained effort. This sequential relationship highlights the progression from awareness to ethical commitment and behavioral consistency in promoting sustainability within healthcare settings [13].

Accordingly, this study aims to examine the relationships between nurses’ sustainability consciousness, green advocacy, and work grit within healthcare settings. Specifically, it assesses nurses’ levels of sustainability consciousness, green advocacy, and work grit, examines the associations among these variables, and determines the extent to which sustainability consciousness and work grit predict green advocacy behaviors. These research objectives address key questions regarding how awareness and psychological resilience influence environmentally responsible practices among nurses. Understanding these relationships is essential for informing workforce development strategies, organizational policies, and interventions that support environmentally sustainable healthcare systems and enhance public health outcomes.

## 2. Theoretical Framework

This study is grounded in the Theory of Planned Behavior (TPB) and Social Cognitive Theory (SCT) to provide a clear conceptual basis for examining the association among sustainability consciousness, work grit, and green advocacy among nurses.

According to TPB, behavior is influenced by individuals’ attitudes, subjective norms, and perceived behavioral control. In the context of nursing, positive attitudes toward sustainability, supportive professional norms, and a strong sense of control over environmental actions are expected to promote engagement in green advocacy behaviors.

SCT emphasizes the reciprocal interaction between personal factors, behavior, and the environment. Nurses’ sustainability consciousness (personal factor) interacts with their work grit (psychological resource) and organizational environment to facilitate sustained engagement in environmentally responsible practices. SCT thus highlights how individual knowledge and resilience, combined with environmental cues and social reinforcement, support continuous green advocacy.

Integrating these theories, this study hypothesizes that:

1. Higher sustainability consciousness predicts greater green advocacy.
2. Work grit strengthens the translation of sustainability awareness into persistent advocacy behaviors.
3. Organizational and social environments moderate the effectiveness of nurses’ pro-environmental actions.

This theoretical grounding ensures that the study’s variables are conceptually linked and provides a framework for interpreting the observed associations.

To strengthen the research gap, we highlight the critical role of Work Grit in nursing practice. While Sustainability Consciousness reflects a nurse’s intention and motivation to engage in environmentally friendly practices, Work Grit provides the persistence, resilience, and determination needed to translate these intentions into consistent action, even in challenging hospital environments. By linking Sustainability Consciousness with Work Grit, this study examines how both constructs jointly influence nurses’ Green Advocacy behaviors, offering a more complete understanding of the drivers of sustainable healthcare practices.

## 3. Materials and Methods

### 3.1. Study Design

A descriptive cross-sectional study was conducted. Due to the cross-sectional nature of this study, the findings reflect associations between variables rather than causal relationships.

### 3.2. Study Setting

The study was conducted at two university-affiliated hospitals in Egypt: Al-Rajhi University Hospital (200 beds) and the Heart University Hospital (240 beds), both affiliated with Assiut University. These hospitals provide a range of medical and specialized services and employ nurses across diverse clinical units.

### 3.3. Participants

The study population comprised all registered nurses working at the two selected hospitals during the data collection period. A census sampling approach was adopted, whereby all eligible nurses were invited to participate. A total of 377 nurses consented and completed the study questionnaires (Al-Rajhi Hospital: 127 nurses; Heart Hospital: 250 nurses).

Inclusion criteria included being a registered nurse at one of the selected hospitals and willingness to participate in the study. Nurses on extended leave during the data collection period were excluded.

“It acknowledged that this study was conducted in only two university hospitals in Upper Egypt. Therefore, the findings may not fully generalize to nurses working in other regions, private hospitals, or different healthcare settings.”

### 3.4. Data Collection Instruments

Data were collected using a structured, self-administered questionnaire consisting of four sections:

#### 3.4.1. Personal Characteristics

This section included items such as hospital affiliation, age, gender, marital status, educational qualification, and years of professional experience.

#### 3.4.2. Sustainability Consciousness

Sustainability consciousness was measured using the Sustainability Consciousness Questionnaire—Short Version (SCQ-S), developed by Gericke et al. [14]. The instrument comprises 27 items covering three dimensions: sustainability knowingness, attitudes, and behaviors across environmental, social, and economic domains. Items were rated on a three-point Likert scale (1 = Agree, 2 = Neutral, 3 = Disagree). Total scores range from 27 to 81, with higher scores indicating higher levels of sustainability consciousness.

For interpretation purposes, total scores were categorized into “High,” “Moderate,” and “Low” levels based on Best’s criteria: scores above 75% were classified as High, scores between 50 and 75% were classified as Moderate, and scores below 50% were classified as Low.

#### 3.4.3. Green Advocacy

Green advocacy was assessed using a modified version of the Green Advocacy Scale developed by Kim et al. [15]. The scale consists of three items evaluating the extent to which nurses encourage and influence colleagues to engage in environmentally sustainable practices within the workplace. Responses were rated on a five-point Likert scale ranging from 1 (Strongly disagree) to 5 (Strongly agree). Higher mean scores indicate greater engagement in green advocacy behaviors.

Green advocacy scores were also categorized into “High,” “Moderate,” and “Low” using the same thresholds (above 75% = High; 50–75% = Moderate; below 50% = Low) to facilitate interpretation and comparison.

#### 3.4.4. Work Grit

Work grit was measured using the 12-item Grit Scale developed by Duckworth et al. [16], which assesses perseverance and passion for long-term goals. Items were rated on a three-point Likert-type scale (1 = Not like me, 2 = Somewhat like me, 3 = Very much like me). Six items were reverse-scored. Mean scores range from 1 to 3, with higher scores indicating greater work grit.

### 3.5. Validity and Reliability

The study instruments were translated into Arabic using a forward–backward translation procedure to ensure linguistic and conceptual equivalence. Face and content validity were assessed by a panel of five experts in nursing administration and healthcare sustainability. Reliability was evaluated using Cronbach’s alpha coefficients, which demonstrated good internal consistency for all scales: sustainability consciousness (α = 0.852), green advocacy (α = 0.882), and work grit (α = 0.788).

### 3.6. Pilot Study

A pilot study was conducted with 10% of the total sample (n = 37 nurses) to assess the clarity, feasibility, and time required to complete the questionnaire. Participants included in the pilot study were excluded from the final analysis. Minor wording adjustments were made based on pilot feedback.

### 3.7. Data Collection Procedure

Data were collected over a three-month period from September to November 2025. Questionnaires were distributed to eligible nurses during working hours after a brief explanation of the study objectives. Participation was voluntary, and completed questionnaires were returned directly to the researchers. No identifying information was collected.

### 3.8. Ethical Considerations

The study was conducted in accordance with the Declaration of Helsinki and was approved by the Ethics Committee of the Faculty of Nursing, Assiut University (Approval No. 1120261382; Date: 29 March 2025). Participants were informed about the study objectives, confidentiality of data, and their right to withdraw at any time without penalty. Written informed consent was obtained from all participants prior to data collection.

### 3.9. Statistical Analysis

Data were analyzed using IBM SPSS Statistics version 20.0. Data normality was assessed using the Anderson–Darling test, and homogeneity of variance was evaluated prior to inferential analysis. Descriptive statistics were used to summarize categorical variables as frequencies and percentages, and continuous variables as means and standard deviations.

Pearson’s correlation coefficients were calculated to examine associations between sustainability consciousness, green advocacy, and work grit. Independent-samples *t*-tests and one-way analysis of variance (ANOVA) were used to compare mean scores across demographic variables.

Multiple linear regression analysis was performed to identify predictors of green advocacy. Variables were selected for the regression model based on their performance in the initial correlation analysis (*p* < 0.20). Multicollinearity was assessed using the Variance Inflation Factor (VIF) and tolerance values; all VIF values were below 5, and tolerance values were above 0.2, indicating no multicollinearity concerns. The “Enter” method was used to enter variables into the model, allowing evaluation of the incremental contribution of Work Grit beyond Sustainability Consciousness and demographic factors. Assumptions of linearity, normality, and homoscedasticity of residuals were verified. A two-tailed *p*-value < 0.05 was considered statistically significant.

To address the high correlations observed among the three dimensions of sustainability consciousness (r > 0.80), a composite Sustainability Index was created by averaging the scores across the knowingness, attitudes, and behaviors subscales. This index was then used in the regression model to represent overall sustainability consciousness, reducing potential overlap among highly correlated predictors and improving model stability.

## 4. Results

### 4.1. Participant Characteristics

A total of 377 nurses participated in the study. The majority were female and aged between 25 and 35 years, with most holding a diploma or bachelor’s degree in nursing. Nearly half of the participants had between one and five years of professional experience. Detailed demographic characteristics are presented in Table 1.

### 4.2. Sustainability Consciousness Among Nurses

Nurses demonstrated generally high levels of sustainability consciousness across environmental, social, and economic dimensions (Table 2). Among the three components, sustainability knowingness showed the highest mean scores, followed by attitudes and behaviors. While overall sustainability consciousness was high, comparatively lower mean scores were observed for environmental attitudes and economic behaviors, indicating potential variability in how sustainability knowledge is applied in practice.

**Figure 1:**
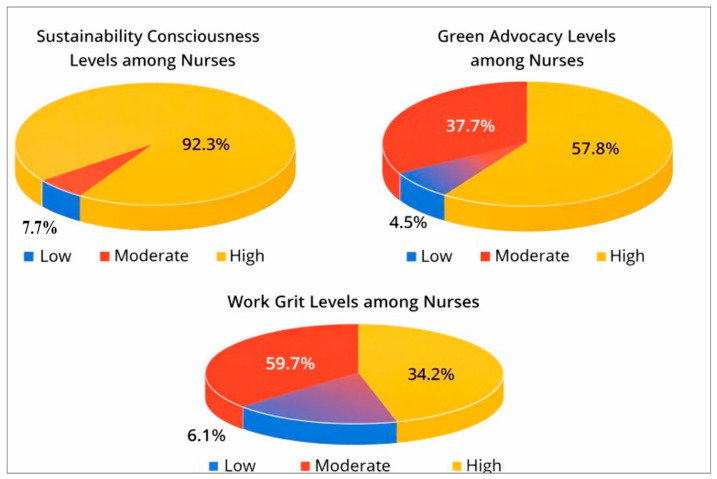
Sustainability Consciousness Levels, Green Advocacy Levels, and Work Grit Levels among Nurses.

As illustrated in Figure 1, the majority of nurses were classified as having a high level of sustainability consciousness, with only a small proportion exhibiting moderate or low levels. These results describe associations between knowledge, attitudes, and behaviors, without implying causation.

### 4.3. Green Advocacy and Work Grit Levels

More than half of the nurses demonstrated high levels of green advocacy, while a considerable proportion exhibited moderate levels, and only a small percentage reported low engagement (Figure 1). Overall, green advocacy scores indicated a moderate-to-high level of engagement among participants.

At the item level, the three statements assessing green advocacy reflected nurses’ willingness to encourage colleagues, support environmentally responsible practices, and promote sustainability within the workplace. Although the scale is brief, responses showed a generally consistent pattern of positive engagement across participants.

Regarding work grit, most nurses reported moderate levels, followed by those with high levels of grit (Figure 1). A limited proportion of participants exhibited low work grit, suggesting generally adequate levels of perseverance and commitment within the nursing workforce. These findings reflect observed associations and distributions rather than causal relationships.

### 4.4. Associations Among Sustainability Consciousness, Green Advocacy, and Work Grit

Pearson correlation analysis revealed statistically significant positive associations among sustainability consciousness, green advocacy, and work grit (Table 3). Sustainability consciousness and its dimensions were strongly interrelated. Green advocacy showed moderate positive associations with overall sustainability consciousness and sustainability behaviors. Work grit demonstrated weak-to-moderate positive associations with sustainability consciousness and a moderate association with green advocacy.

**Figure 2:**
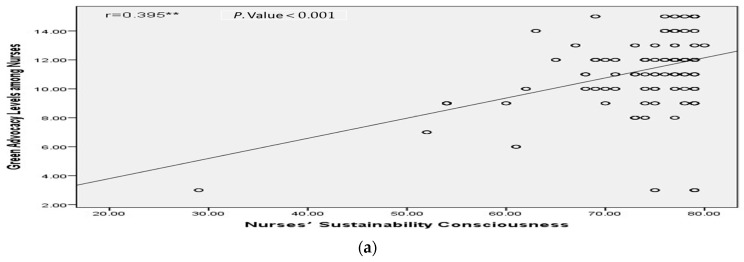

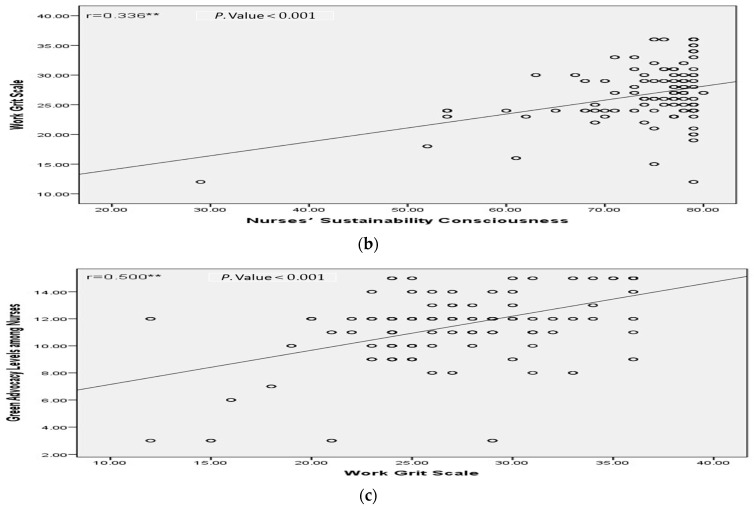
Scatter plots illustrating the associations among sustainability consciousness, work grit, and green advocacy among nurses. (**a**) The association between sustainability consciousness and green advocacy; (**b**) The association between sustainability consciousness and work grit; (**c**) The association between work grit and green advocacy. ** *p* < 0.001; Circles represent individual data points for nurses included in the study.

Figure 2 further illustrate these significant positive associations, highlighting the interconnected nature of sustainability awareness, advocacy behaviors, and perseverance among nurses.

### 4.5. Differences According to Personal Characteristics

Significant associations were observed between sustainability consciousness, green advocacy, and several demographic variables (Table 4). Nurses working at the Heart University Hospital reported higher levels of sustainability consciousness and green advocacy compared with those at Al-Rajhi Hospital. Age was significantly associated with all three study variables, with younger nurses showing higher sustainability consciousness and green advocacy, whereas older nurses showed higher work grit.

Gender differences were observed only for sustainability consciousness, while marital status was associated with sustainability consciousness and green advocacy but not with work grit. Higher educational qualifications were consistently associated with greater sustainability consciousness, green advocacy, and work grit. Years of professional experience were also associated with all three variables, with nurses in early career stages showing higher sustainability consciousness and advocacy, and those with longer experience showing higher work grit.

### 4.6. Predictors of Green Advocacy

Multiple linear regression analysis showed that sustainability consciousness and work grit were significantly positively associated with green advocacy (Table 5). Educational qualification was also positively associated, while age was significantly negatively associated. The overall regression model was statistically significant and explained approximately one-third of the variance in green advocacy among nurses. Regression diagnostics indicated no multicollinearity issues (VIF < 5, tolerance > 0.2), and the assumptions of linearity, normality, and homoscedasticity of residuals were satisfied, supporting the validity of the model.

## 5. Discussion

The present study provides empirical evidence on the relationships between sustainability consciousness, green advocacy, and work grit among nurses. The findings contribute to improving the understanding of factors that influence environmentally sustainable behaviors in healthcare settings, with important implications for both organizational practice and public health.

The results demonstrated that nurses reported high levels of sustainability consciousness across environmental, social, and economic dimensions, indicating a strong awareness of sustainability principles relevant to healthcare delivery. From a systems perspective, such awareness represents a foundational human resource for sustainability transitions, as individual knowledge and attitudes are necessary precursors for collective and organizational change. Similar findings have been reported in previous studies showing that healthcare professionals increasingly recognize sustainability as integral to quality care, particularly in relation to resource efficiency and environmental responsibility [17,18].

In addition to high sustainability consciousness, a substantial proportion of nurses demonstrated moderate to high levels of green advocacy. This suggests that sustainability awareness among nurses extends beyond personal behaviors to include efforts aimed at influencing colleagues, routines, and workplace practices. Green advocacy is especially critical in healthcare systems, where environmental improvements often require coordinated action across teams and units rather than isolated individual behaviors. Consistent with previous research, nurses with higher sustainability awareness were more likely to engage in advocacy behaviors that promote waste reduction, energy conservation, and environmentally responsible decision-making within organizational contexts, while also addressing issues such as workplace safety and violence [19,20].

Work grit emerged as an important psychological resource in this study, with most nurses demonstrating moderate levels of perseverance and commitment. Healthcare environments are characterized by high workloads, emotional demands, and operational constraints, all of which can impede sustained engagement in sustainability initiatives. The observed levels of work grit suggest that many nurses possess the resilience necessary to remain engaged with long-term goals despite these challenges. This finding aligns with existing evidence identifying grit as a supportive resource for sustained motivation, adaptive coping, and professional engagement in demanding healthcare settings [21,22].

The correlation analysis revealed significant positive relationships among sustainability consciousness, green advocacy, and work grit. These findings indicate that nurses who are more aware of sustainability issues are also more likely to demonstrate higher perseverance and engage in advocacy behaviors. Importantly, the association between work grit and green advocacy suggests that resilience and persistence may facilitate the translation of sustainability awareness into sustained advocacy actions within complex organizational environments. From a sustainability transition perspective, this supports the notion that cognitive awareness alone is insufficient; sustained pro-environmental engagement also requires psychological resources that enable individuals to navigate resistance, workload pressures, and institutional constraints, as evidence indicates that structured lifestyle modifications can significantly reduce disease severity and operative outcomes when individuals are supported and monitored over time [23].

The multiple linear regression analysis further confirmed that sustainability consciousness and work grit are significant predictors of green advocacy, collectively explaining a meaningful proportion of variance in advocacy behaviors. This finding underscores the complementary roles of cognitive and psychological factors in supporting sustainable healthcare practices. Nurses who possess both sustainability awareness and perseverance appear better equipped to contribute to environmentally responsible practices and influence organizational change. These results are consistent with sustainability behavior frameworks that emphasize the interaction between knowledge, motivation, and personal resilience in sustaining pro-environmental actions over time [24].

The negative association between age and green advocacy observed in this study may reflect generational differences in sustainability education and exposure to contemporary environmental discourse. Younger nurses may have benefited from more recent curricular emphasis on sustainability and climate-related health issues, supporting their engagement in advocacy behaviors. In contrast, older nurses, while demonstrating higher levels of work grit, may have had fewer opportunities for structured sustainability training. This finding highlights the importance of continuous professional development strategies that strengthen sustainability competencies across all career stages.

Educational qualification emerged as a positive predictor of green advocacy, suggesting that higher levels of academic preparation may enhance nurses’ capacity for critical thinking, systems awareness, and advocacy related to sustainability. This reinforces the role of education as a key mechanism for strengthening organizational sustainability capacity, particularly through integrating sustainability and environmental health content into nursing education and continuing professional development programs [24].

From a policy and organizational sustainability perspective, these findings have important implications. Strengthening nurses’ sustainability consciousness through education and institutional support, while simultaneously fostering work grit through supportive and enabling work environments, may enhance green advocacy and accelerate sustainability transitions within healthcare systems. Nurses’ advocacy behaviors have the potential to influence not only daily clinical practices but also organizational norms, policies, and long-term sustainability strategies, thereby supporting healthcare systems that are more resilient, resource-efficient, and aligned with global sustainability goals SDG 13 (climate action) and SDG 3 (health and well-being) [25].

The most effective path to sustainable healthcare involves integrating regulatory strategies with initiatives that enhance sustainability consciousness among healthcare professionals. Regulations establish the structural conditions for change, while sustainability consciousness and organizational culture drive the behavioral adoption necessary to ensure that sustainable practices are durable and effective [26,27].

This study contributes to sustainability science by extending understanding of how human and psychological factors support sustainability transitions within healthcare systems. By integrating sustainability consciousness, green advocacy, and work grit, the study highlights the role of frontline healthcare professionals as agents of organizational sustainability and demonstrates how individual resilience resources can facilitate the translation of sustainability awareness into sustained advocacy and system-level changes in resource-intensive healthcare environments.

Collectively, these findings underscore the interconnected roles of sustainability awareness, advocacy behaviors, and personal resilience in advancing environmentally sustainable healthcare practices, while simultaneously fostering adaptive behaviors, collaboration, and readiness for change within supportive learning and organizational environments that encourage participation, innovation, and professional engagement among nurses [28,29,30]. Strengthening these factors among nurses may support healthcare systems in becoming more resilient, resource-efficient, and aligned with global sustainability priorities and the Sustainable Development Goals (SDG 3; Good Health and Well-Being).

Overall, the study strengthens the evidence base for integrating sustainability awareness and psychological resilience into healthcare workforce development to promote sustainable and high-quality care.

### Study Limitations

This study has several limitations that should be considered when interpreting the findings. First, the cross-sectional design provides a snapshot in time, which means causal relationships between Work Grit and Green Advocacy cannot be established—only associations are observed. Second, data were collected using self-reported questionnaires, which may be influenced by social desirability bias; participants might have reported being more environmentally engaged than in practice. Future research could address these limitations by employing longitudinal designs, including objective measures of green behaviors, and expanding the sample to other healthcare settings to enhance generalizability. Additionally, green advocacy was measured using a three-item scale, which, although reliable, may limit the comprehensiveness of the construct assessment.

## 6. Conclusions

This study provides empirical evidence of significant positive relationships among nurses’ sustainability consciousness, green advocacy, and work grit within healthcare settings. Nurses with higher levels of sustainability consciousness were more likely to engage in green advocacy, indicating that sustainability awareness is a key driver of environmentally responsible practices in healthcare organizations. In addition, sustainability consciousness was positively associated with work grit, suggesting that nurses who demonstrate greater perseverance and commitment are also more attuned to sustainability principles.

The positive association between work grit and green advocacy highlights the importance of psychological resilience in sustaining pro-environmental engagement within complex and resource-intensive healthcare systems. From a systems perspective, these findings suggest that individual sustainability awareness must be supported by personal and organizational resources to translate into sustained advocacy and meaningful practice change. Nurses who possess both sustainability consciousness and perseverance may be better equipped to navigate organizational barriers, maintain long-term engagement, and contribute to sustainability transitions within healthcare systems.

Collectively, these findings underscore the interconnected roles of sustainability awareness, advocacy behaviors, and personal resilience in advancing environmentally sustainable healthcare practices. Strengthening these factors among nurses may support healthcare systems in becoming more resilient, resource-efficient, and aligned with global sustainability priorities and the Sustainable Development Goals.

## 7. Practical Implications and Recommendations

Based on the study findings, several practical and policy-oriented recommendations can be proposed:

Healthcare organizations should integrate sustainability education into ongoing professional development programs to strengthen nurses’ sustainability consciousness and promote consistent engagement in environmentally responsible practices. Clear institutional policies and operational guidelines supporting waste reduction, energy conservation, and sustainable resource use should be developed and actively communicated to encourage nurses’ participation in sustainability initiatives.

Supportive organizational climates that recognize and reinforce pro-environmental behaviors should be fostered to enhance nurses’ perseverance, motivation, and long-term commitment to sustainability goals. Nursing leadership should actively model sustainability-oriented behaviors and green advocacy, thereby reinforcing a culture of environmental responsibility across healthcare teams.

Future research should employ longitudinal or intervention-based designs to explore causal relationships among sustainability consciousness, work grit, and green advocacy, and evaluate the effectiveness of targeted strategies aimed at strengthening sustainable healthcare practices.

From a policy perspective, the findings highlight the need for a more structured integration of sustainability into nursing systems and evaluation frameworks. Healthcare organizations should consider embedding “green nursing” indicators into performance appraisal systems and clinical competency frameworks to ensure that environmentally responsible practices are consistently monitored and reinforced. Incorporating sustainability metrics into quality assurance and accreditation standards may further institutionalize environmentally sustainable behaviors within routine clinical practice.

In addition, work grit should not be viewed solely as a fixed individual trait, but rather as a developable professional capacity. Organizational strategies such as resilience training programs, mentorship initiatives, and supportive leadership practices may help strengthen nurses’ grit over time. Enhancing work grit may not only promote sustained engagement in green advocacy but also contribute to reducing burnout and improving workforce retention, particularly in high-demand healthcare environments. Thus, integrating sustainability-focused policies with psychological resource development may offer a comprehensive approach to advancing sustainable healthcare systems.

## Figures and Tables

**Table 1:** Demographic characteristics of participating nurses (n = 377).

Variable	Category	n (%)
Hospital	Al-Rajhi Assiut University Hospital	141 (37.4)
	Heart Assiut University Hospital	236 (62.6)
Age (years)	25–<35	267 (70.8)
	36–<45	92 (24.4)
	46–<55	18 (4.8)
Gender	Male	74 (19.6)
	Female	303 (80.4)
Marital status	Single	152 (40.3)
	Married	216 (57.3)
	Divorced	3 (0.8)
	Widowed	6 (1.6)
Educational qualification	Nursing institute	188 (49.9)
	Bachelor’s degree in nursing	174 (46.2)
	Master’s degree	6 (1.6)
	Doctoral degree	9 (2.4)
Years of experience	1–<5	172 (45.6)
	5–<10	130 (34.5)
	10–<15	48 (12.7)
	≥15	27 (7.2)

Note: n = sample size; n (subcategory) = subgroup frequency; % = percentage.

**Table 2:** Mean scores of nurses’ sustainability consciousness dimensions (n = 377).

Dimension	Subdimension	Maximum Score	Mean ± SD	Range	Mean (%)
Sustainability Knowingness	Environmental	9	8.75 ± 0.81	3–9	97.3
	Social	9	8.65 ± 0.88	3–9	96.1
	Economic	9	8.51 ± 1.05	3–9	94.5
	Total	27	25.91 ± 2.46	9–27	96.0
Sustainability Attitudes	Environmental	9	7.19 ± 0.74	5–9	79.9
	Social	9	8.48 ± 1.02	3–9	94.3
	Economic	9	8.50 ± 1.02	3–9	94.4
	Total	27	24.17 ± 2.07	11–27	89.5
Sustainability Behaviors	Environmental	9	8.41 ± 1.17	3–9	93.5
	Social	9	8.44 ± 1.13	3–9	93.8
	Economic	9	7.80 ± 1.41	3–9	86.6
	Total	27	24.65 ± 3.25	9–27	91.3
Overall Sustainability Consciousness	—	81	74.73 ± 6.91	29–80	92.3

Note: n = sample size; SD = standard deviation.

**Table 3:** Pearson correlation matrix among study variables (n = 377).

Variable	1	2	3	4	5
1. Sustainability Knowingness	1				
2. Sustainability Attitudes	0.803 **	1			
3. Sustainability Behaviors	0.612 **	0.660 **	1		
4. Nurses’ Sustainability Consciousness	0.884 **	0.896 **	0.886 **	1	
5. Green Advocacy Levels among Nurses	0.287 **	0.261 **	0.457 **	0.395 **	1
6. Work Grit	0.284 **	0.297 **	0.311 **	0.336 **	0.500 **

Pearson correlation coefficients are shown below the diagonal. ** *p* < 0.01.

**Table 4:** Comparison of nurses’ sustainability consciousness, green advocacy, and work grit across personal characteristics (n = 377).

Characteristic	n	Sustainability Consciousness (Mean ± SD)	Green Advocacy (Mean ± SD)	Work Grit (Mean ± SD)
Hospital				
Al-Rajhi Assiut University Hospital	141	73.51 ± 8.65	10.95 ± 2.45	26.33 ± 4.64
Heart Assiut University Hospital	236	75.46 ± 5.51	11.69 ± 2.38	27.22 ± 4.89
Test (*p*)		t = 7.12 (0.008 **)	t = 8.35 (0.004 **)	t = 3.09 (0.079)
Age (years)				
25–<35	267	75.64 ± 4.43	11.65 ± 2.12	26.73 ± 4.33
36–<45	92	73.09 ± 10.61	11.07 ± 3.15	26.78 ± 5.87
46–<55	18	69.67 ± 9.57	9.67 ± 1.64	29.83 ± 4.91
Test (*p*)		F = 10.21 (<0.001 **)	F = 7.10 (0.001 **)	F = 3.59 (0.028 *)
Gender				
Male	74	76.42 ± 4.14	11.62 ± 2.62	26.07 ± 5.27
Female	303	74.32 ± 7.38	11.36 ± 2.38	27.09 ± 4.68
Test (*p*)		t = 5.57 (0.019 *)	t = 0.67 (0.413)	t = 2.69 (0.102)
Marital status				
Single	152	76.08 ± 4.19	11.92 ± 2.18	26.76 ± 4.80
Married	216	73.87 ± 8.10	11.07 ± 2.57	27.08 ± 4.90
Divorced	3	60.00 ± 0.00	9.00 ± 0.00	24.00 ± 0.00
Widowed	6	79.00 ± 0.00	12.00 ± 0.00	24.50 ± 0.55
Test (*p*)		F = 8.89 (<0.001 **)	F = 4.86 (0.002 **)	F = 1.01 (0.390)
Education				
Nursing institute	188	73.63 ± 8.86	10.94 ± 2.05	26.43 ± 4.52
Bachelor’s degree	174	75.55 ± 3.92	11.71 ± 2.69	26.78 ± 4.82
Master’s degree	6	79.00 ± 0.00	15.00 ± 0.00	36.00 ± 0.00
Doctoral degree	9	79.00 ± 0.00	13.33 ± 1.32	32.67 ± 2.00
Test (*p*)		F = 4.44 (0.004 **)	F = 18.00 (<0.001 **)	F = 13.29 (<0.001 **)
Years of experience				
1–<5	172	76.42 ± 3.41	11.47 ± 2.25	26.18 ± 4.67
5–<10	130	75.28 ± 5.67	11.79 ± 2.41	27.71 ± 4.13
10–<15	48	70.08 ± 13.00	10.69 ± 3.10	25.40 ± 6.15
≥15	27	69.56 ± 7.81	10.56 ± 1.87	30.11 ± 4.12
Test (*p*)		F = 18.18 (<0.001 **)	F = 0.71 (0.012 *)	F = 8.56 (<0.001 **)

Notes: Data are presented as mean ± SD. Independent *t*-tests were used for two-group comparisons; one-way ANOVA was used for three or more groups. * *p* < 0.05; ** *p* < 0.01.

**Table 5:** Multiple linear regression analysis predicting green advocacy among nurses (n = 377).

Predictor	B	SE	t	*p*	β	95% CI for B
Nurses’ Sustainability Consciousness	0.07	0.02	4.13	<0.001 **	0.19	0.04–0.1
Work Grit	0.22	0.02	9.35	<0.001 **	0.43	0.17–0.26
Age	−0.74	0.19	−3.95	<0.001 **	−0.17	−1.11 to −0.37
Educational qualification	0.41	0.16	2.53	0.012 *	0.11	0.09–0.73

Model statistics: R^2^ = 0.342; F = 49.79; *p* < 0.001; Dependent variable: green advocacy. Notes: B = unstandardized regression coefficient; β = standardized coefficient; SE = standard error; CI = confidence interval; * *p* < 0.05; ** *p* < 0.01.

## Data Availability

Data is contained within the article.

## Abbreviations

The following abbreviations are used in this manuscript:

SDGs	Sustainable Development Goals
SCQ	Sustainability Consciousness Questionnaire
